# Comprehensive Identification and Male-Biased Expression Analysis of Odorant-Binding Protein Genes in the Hawaiian Flower Thrips, *Thrips hawaiiensis* (Thysanoptera: Thripidae)

**DOI:** 10.3390/biology15020170

**Published:** 2026-01-17

**Authors:** Qingqing Fan, Yanjun Li, Xiaodi Hu

**Affiliations:** State Key Laboratory for Quality and Safety of Agro-Products, Key Laboratory of Biotechnology in Plant Protection of MARA, Zhejiang Key Laboratory of Green Plant Protection, Institute of Plant Virology, Ningbo University, Ningbo 315211, China; 13173901995@163.com (Q.F.); liyanjun@nbu.edu.cn (Y.L.)

**Keywords:** *Thrips hawaiiensis*, odorant-binding proteins, RT-qPCR, genome-wide analysis, chemosensory proteins

## Abstract

Olfaction is essential for key insect behaviors, such as host-seeking and mating, and is initiated by odorant-binding proteins (OBPs), which bind and transport hydrophobic odors. *Thrips hawaiiensis* is a major pest that infests the flowers of numerous horticultural crops, yet its chemosensory mechanisms remain poorly understood. Now, the availability of its genome assembly allows us to address this gap. To this end, we performed a comprehensive exploration and comparative analysis of its OBP genes. Our genome-wide analysis identified a total of 12 OBP genes in *T. hawaiiensis*, whereas the repertoire across other published thrips genomes ranges from 10 to 17, a count significantly lower than that in most other insects. Notably, transcriptomic and RT-qPCR analyses revealed consistent male-biased expression of OBPs in *T. hawaiiensis*, supporting their role in mate-finding and foraging behaviors. Furthermore, we identified 11 chemosensory proteins (CSPs). Transcriptomic and RT-qPCR analyses revealed that these CSPs exhibit an expression pattern similar to that of the OBPs, with over half of the genes showing significantly higher expression in males. This work provides a foundational framework for future functional studies of olfactory proteins, both in *T. hawaiiensis* and the wider insect community.

## Simple Summary

The Hawaiian flower thrips, *Thrips hawaiiensis*, is a widespread pest that infests the flowers of numerous horticultural crops. We characterized the previously unknown olfactory system of *Thrips hawaiiensis* by analyzing its odorant-binding proteins (OBPs). Our genome survey revealed 12 OBP genes, a count comparable to other thrips but low relative to most insects. Subsequent transcriptomic and RT-qPCR analyses identified consistent male-biased expression, implicating these OBPs in male-specific olfactory behaviors, such as mate location and foraging. We further identified 11 CSPs, the majority of which showed a male-biased expression pattern similar to that of the OBPs.

## 1. Introduction

*Thrips hawaiiensis* (Morgan) (Thysanoptera: Thripidae), a common flower-dwelling thrips species native to the Oriental and Pacific regions, has expanded its geographical range through international trade to Africa, Australia, Europe, and the Americas [1,2,3,4,5]. *T. hawaiiensis* primarily feeds and resides within floral tissues [1,5,6]. The elevated risk of global introduction and spread of *T. hawaiiensis*, driven by its strong thigmokinetic behavior, high fecundity, and short life cycle [1,7], is fueling growing concern about its potential to become a major pest of many crops. As a strictly flower-dwelling species, *T. hawaiiensis* inflicts damage on a wide range of crops, including tobacco [8], rose [9], gladiolus [10], *Brassica oleracea* [11], coffee [12], mango [13], citrus [14], apples and pears [6], and bananas [15,16]. Direct feeding on floral and fruit tissues induces symptoms such as scarring, necrosis, and malformation, the severity of which depends on infestation intensity. Furthermore, its pollen-feeding behavior can adversely affect plant fertility [17].

Olfaction is essential for detecting and interpreting environmental cues, enabling organisms to locate food, hosts, mates, and oviposition sites, as well as to evade predators and pathogens [18]. Both invertebrates and vertebrates use the olfactory system to mediate odorant detection [19]. The accurate operation of the olfactory system depends on a coordinated series of olfactory proteins. Key among these are odorant-binding proteins (OBPs), chemosensory proteins (CSPs), odorant receptors (ORs), gustatory receptors (GRs), ionotropic receptors (IRs), sensory neuron membrane proteins (SNMPs), odorant-degrading enzymes (ODEs), and Niemann–Pick protein C2 (NPC2) [20,21]. Notably, OBPs participate in the initial biochemical reaction of the olfactory transduction cascade. They play a critical role by discriminating, binding, and transporting odorant molecules to their corresponding Ors [22,23,24]. OBPs are characterized as small, water-soluble, and extracellular, residing in the sensillar lymph that bathes the sensory dendrites [19,25]. The classification of OBPs is based on their conserved cysteine patterns, yielding distinct subfamilies: Classic (six cysteines), Minus-C (four or five), Plus-C (at least eight), and Dimer/Atypical OBPs, the latter two comprising tandem or fused classic domains that may form dimer-like structures [23,26,27,28]. The first OBP was identified in *Antheraea polyphemus* [29]. Subsequently, transcriptomic and genomic data have facilitated the identification of a rapidly increasing number of OBPs. Although OBPs have been characterized in several thrips species, including *Frankliniella occidentalis* [30], *Frankliniella intonsa* [30], and *Megalurothrips usitatus* [26], this gap persists for *Thrips hawaiiensis*. The recent availability of a genome assembly for *T. hawaiiensis* [31] provides a crucial resource to address this gap, allowing for a comprehensive exploration and comparative analysis of its OBP repertoire.

We conducted a comprehensive analysis of odorant-binding protein (OBP) genes in *Thrips hawaiiensis*, beginning with genome-wide identification. This was followed by characterization of their chromosomal distribution, phylogenetic evolution, gene structures, and protein domains. Tissue-specific expression profiling across both sexes was performed via RT-qPCR. Collectively, our results lay the necessary groundwork for elucidating the functional roles of OBPs and for devising targeted, eco-friendly control methods for this pest.

## 2. Materials and Methods

### 2.1. Identification of OBP Genes in Thrips

The genome assembly, along with its GFF annotation, coding sequence (CDS), and protein sequence files for *T. hawaiiensis*, were obtained from figshare (https://doi.org/10.6084/m9.figshare.26125162) [31]. To investigate the prevalence of OBPs in thrips, we also retrieved publicly available whole-genome data of thrips (as of that available from June 2025), including two other populations of *Megalurothrips usitatus* (samples from Hainan, China [32], and Zhejiang, China [33]), *Dendrothrips minowai* [34], *Frankliniella fusca* [35], *Frankliniella occidentalis* [36], *Odontothrips loti* [37], *Thrips palmi* [38], *Thrips tabaci* [39], *Frankliniella intonsa* [40], and *Stenchaetothrips biformis* [41]. Known OBP amino acid sequences from *Frankliniella intonsa* and *Frankliniella occidentalis* [30] were retrieved from GenBank (National Center for Biotechnology Information, NCBI) and used as query sequences for a BLASTP (v2.5.0) search against the thrips amino acid database, applying an identity cutoff of 30%. Then, gene annotation was performed using hmmscan (v3.1b1) with the Pfam-A.hmm database, applying an E-value threshold of 1 × 10^−5^ to identify putative OBP genes (PF01395.18). To improve accuracy, the hmmscan results were combined with BLAST-based annotations by retaining only overlapping gene predictions, yielding a high-confidence gene set for downstream analyses. The putative N-terminal signal peptides of *T. hawaiiensis* OBPs were identified using the SignalP 6.0 online server (https://services.healthtech.dtu.dk/services/SignalP-6.0/, accessed on 28 October 2025) [42]. The OBP sequences were aligned using MUSCLE (v5.1) [43] software. The resultant alignment file was then imported into Jalview software (v2.11.5.0) [44] for visualization.

### 2.2. Characteristics Analysis of OBPs

The OBP sequences of thrips were aligned using MUSCLE (v5.1) [43]. Following alignment, a maximum likelihood (ML) phylogenetic tree was constructed on the aligned dataset with IQ-TREE (v2.2.5) [45], employing 1000 ultrafast bootstrap replicates (-B 1000). The chromosomal locations of OBP genes were retrieved from the *T. hawaiiensis* GFF annotation file using a custom Python (v3.10.13) script. They were subsequently visualized on the chromosomes via the online tool MapGene2Chrom (http://mg2c.iask.in/mg2c_v2.1/, accessed on 28 October 2025) [46,47]. The exon–intron structures of the ThawOBP genes were determined through an in-house Python script.

### 2.3. Insect Sample Collection

A laboratory population of *Thrips hawaiiensis* was established from individuals collected on kidney beans (*Phaseolus vulgaris*) at the Institute of Plant Protection, Fujian Academy of Agricultural Sciences, China (119°34′ E, 26°13′ N), and has been continuously reared on this host since 2016. The kidney bean diet was prepared by soaking in water, coating with a 10% honey solution, and air-drying. Thrips were maintained in MGC-350HP artificial climate incubators (Yiheng Scientific Instruments, Shanghai, China) under the following conditions: 27 ± 1 °C, 60 ± 5% RH, and a 16:8 (L:D) photoperiod.

### 2.4. RNA Extraction and Real-Time Quantitative PCR Analysis of ThawOBPs

A total of 300 adult male and female *T. hawaiiensis* samples were selected for analysis of relative mRNA expression levels. Total RNA was extracted from the samples using the Trizol method. Subsequently, the RNA was reverse transcribed into cDNA using a reverse transcription kit (Accurate Biology, Changsha, Hunan, China).

A primer design tool (https://www.ncbi.nlm.nih.gov/tools/primer-blast/, accessed on 28 October 2025) was used to design primers for the ThawOBP gene of *Thrips hawaiiensis,* with *β*-actin as the internal reference gene [48]. RT-qPCR was performed on an ABI QuantStudio 5 system (Thermo Fisher Scientific, Waltham, MA, USA), with the entire process conducted on ice. Each experiment included at least two technical replicates and three biological replicates. The expression levels in male and female *Thrips hawaiiensis* were calculated using the 2^−ΔCT^ method. Data analysis and visualization were conducted using GraphPad Prism (v9.5.0) and R (v4.2.0) for correlation calculations. All the primers used in this study are listed in Table 1.

### 2.5. Transcriptome Sequencing

To obtain a comprehensive overview of gene expression, total RNA was isolated from adult male and female subjects. Criteria for cDNA library qualification are as follows: the AD260/280 absorbance ratio should be between 1.8 and 2.0, the A260/230 ratio should be between 1.9 and 2.4, and the concentration measured by Qubit should be between 0.95 and 3.0. Each biological replicate comprised a pool of approximately 800 individuals. RNA extraction was performed using TRIzol Reagent (Thermo Fisher Scientific, USA). Subsequently, Illumina paired-end libraries were constructed with the TruSeq RNA Library Preparation Kit (Illumina, San Diego, CA, USA) according to the manufacturer’s instructions and sequenced on an Illumina NovaSeq 6000 platform. This generated approximately 14.3 GB of high-quality 150 bp paired-end sequence data.

### 2.6. Phylogenetic Analysis of OBP Genes

A maximum likelihood phylogeny was reconstructed using IQ-TREE (v2.3.3) [45]. To determine the most appropriate substitution model, we used the built-in ModelFinder tool, which selected the optimal model from a candidate set based on the Bayesian Information Criterion. The analysis was subsequently conducted under the best-fit model, WAG + R4. Branch support was assessed using ultrafast bootstrap approximation, with 1000 replicates.

## 3. Results

### 3.1. Identification and Sequence Analysis of OBPs in T. hawaiiensis

**Figure 1:**
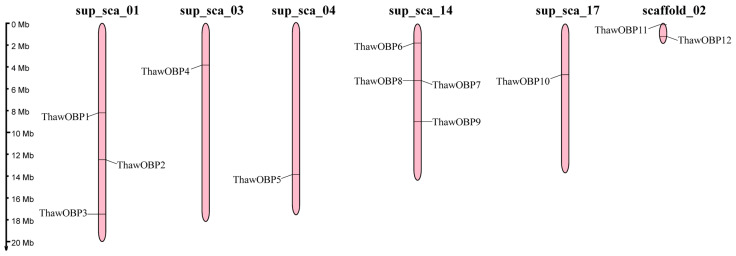
Genomic distribution of ThawOBP genes. Gene positions are indicated along the scale (0–20 Mb).

**Figure 2:**
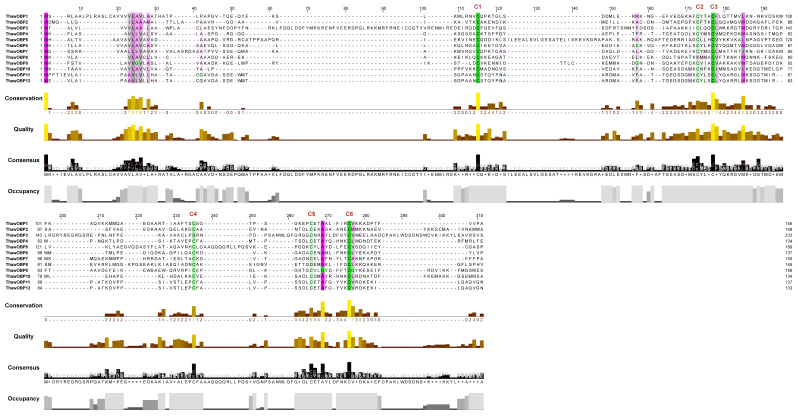
Amino acid sequence alignment of ThawOBPs. Different colors represent different categories of amino acids. In conserved regions, the colors exhibit high consistency, while in variable regions, they appear diverse and multicolored. * Indicates that the amino acid residues at this position are highly conserved.

The genome of T. hawaiiensis was assembled using a hybrid approach that integrated the technologies of Oxford Nanopore long-read sequencing, Illumina short-read sequencing, and Hi-C chromatin conformation capture. This strategy produced a final assembly of 287.59 Mb, with a scaffold N50 of 13.84 Mb. According to BUSCO analysis, the assembled genome exhibits a high completeness of 98.7% [31]. As shown in Table 2, a total of 12 ThawOBP genes were identified through our BLASTP analysis. The genes, named ThawOBP1-ThawOBP12 based on their chromosomal locations (Figure 1), each possessed a complete open reading frame (ORF). The putative proteins range from 133 (ThawOBP12) to 233 (ThawOBP3) amino acids, while most are approximately 150 amino acids long. The analysis classified these genes into two subfamilies: the Classic subfamily (10 genes) and the Minus-C subfamily (two genes, namely ThawOBP3 and ThawOBP8) (Figure 2). All encoded proteins possess a putative N-terminal signal peptide, with the cleavage site predicted between amino acids 17 and 28. These putative ThawOBPs share from 31.4% (ThawOBP2) to 86.8% (ThawOBP5) sequence identity with their closest matches in the database, supported by highly significant E-values ranging from 8.28 × 10^−128^ to 3.67 × 10^−8^ (Table 2). Chromosomal mapping of the 12 identified ThawOBP genes across major genomic scaffolds was conducted. The genes are unevenly distributed, with sup_sca_14 harboring a significant number of ThawOBP loci. Several additional scaffolds also contain ThawOBP genes (Figure 1), suggesting a dispersed genomic organization of the odorant-binding protein family in this species.

### 3.2. Analysis of Phylogenetic Relationship and Gene Structure of OBPs in T. hawaiiensis

**Figure 3:**
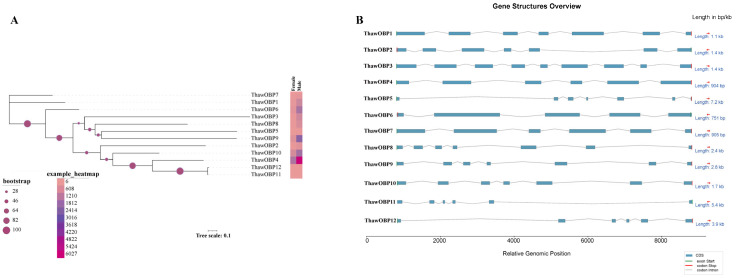
Characteristics of the ThawOBP genes. (**A**) Phylogenetic tree of twelve ThawOBP genes, constructed based on their protein sequences. The tree, with a scale of 0.1, shows bootstrap support values at key nodes. An adjacent heatmap displays the relative expression levels of each ThawOBP gene in adult females and males. (**B**) Schematic overview of the genomic structures of the ThawOBP genes. The diagrams detail the organization of coding sequences (CDS), non-coding regions, exons, start/stop codons, and introns. Red arrows represent the positive and negative strands.

**Figure 4:**
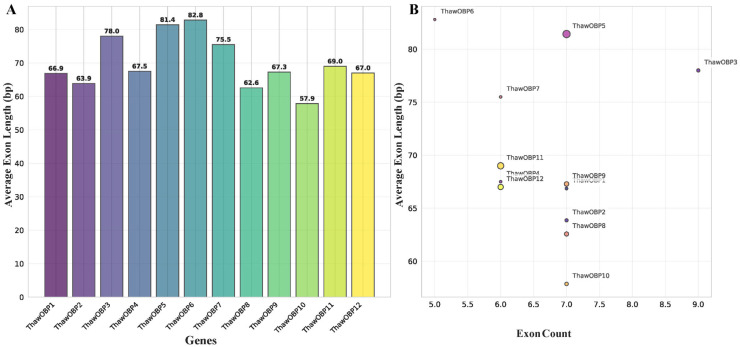
Exon structure characteristics of ThawOBP genes. (**A**) The average exon length across the 12 ThawOBP genes. (**B**) Visual representation of the exon architectural features (count and average length) in the 12 ThawOBP genes. Circle sizes are proportional to gene length, with larger circles representing longer genes.

Investigation of the phylogenetic relationships and exon–intron structures of OBP genes in *T. hawaiiensis* unveiled considerable diversity within this gene family. A phylogenetic tree was constructed from full-length OBP sequences. The tree reveals the evolutionary relationships among the 12 ThawOBP genes, which are categorized into three distinct clades (Figure 3A). ThawOBP11 and ThawOBP12 form a closely related pair with high bootstrap support, suggesting a strong functional constraint. Similarly, ThawOBP4 and ThawOBP10 cluster together, indicating a close evolutionary relationship. ThawOBP1 and ThawOBP7 are positioned more distantly from the other groups, with ThawOBP1 and ThawOBP7 appearing as two of the more divergent sequences in the dataset (Figure 3A). The number of exons in these OBP genes varied between five and nine, with the majority containing six or seven (Figure 3B). Our analysis of the ThawOBP family showed that the average exon length was 69.97 bp. Furthermore, we found that the exon lengths across all 12 genes were relatively constrained, ranging from 57.9 to 82.8 bp (Figure 4A). Among the 12 ThawOBP genes, ThawOBP10 possessed the shortest average exon length (57.9 bp) and contained 7 exons. In contrast, ThawOBP3 had the highest exon count (9) and an average exon length of 78.0 bp. Furthermore, ThawOBP4, ThawOBP11, and ThawOBP12 shared an identical exon number of 6 and exhibited similar average exon lengths (Figure 4B). These structural features are entirely consistent with the clustering pattern observed in Figure 2.

### 3.3. Phylogenetic Relationship Analysis of All OBPs

**Figure 5:**
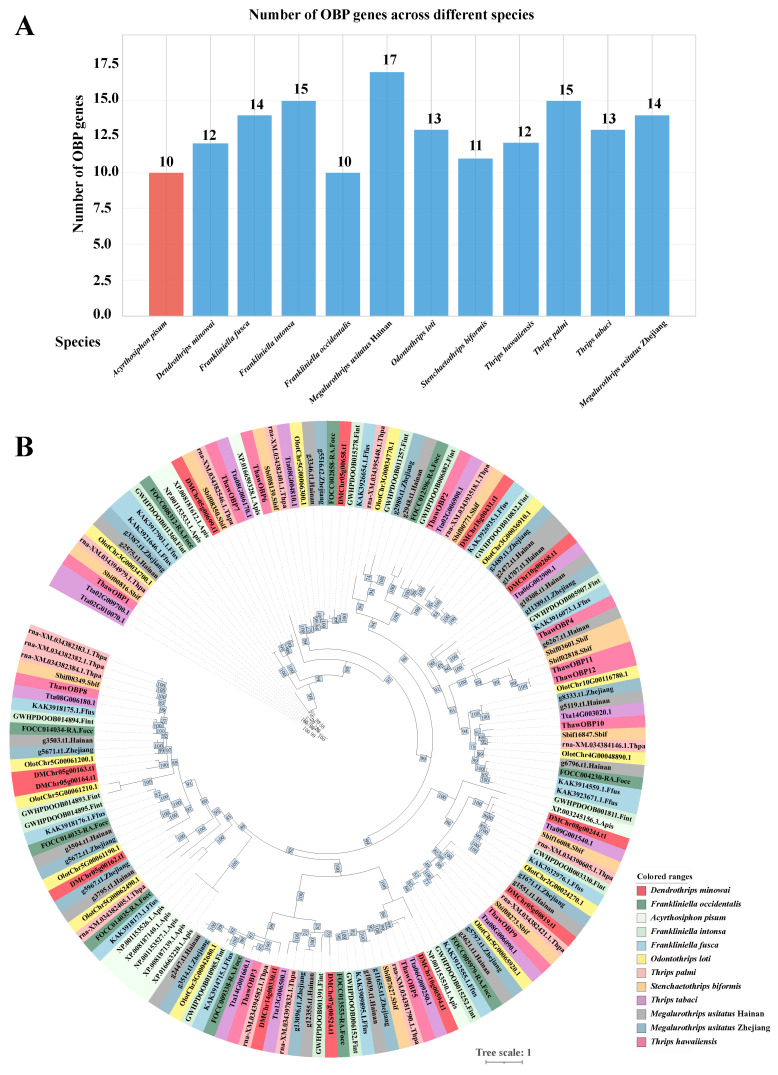
Gene number comparison and phylogenetic analysis of odorant-binding proteins (OBPs) across multiple insect species. (**A**) The total number of OBP genes identified in insect species. The number of OBP genes in *Acyrthosiphon pisum* is represented by red bars, while that in thrips is represented by blue bars. (**B**) A maximum likelihood phylogenetic tree of OBP amino acid sequences. The tree includes representatives from various thrips species and the pea aphid (*Acyrthosiphon pisum*). Colored arcs highlight the clustering of sequences from specific species. Key nodes are numerically labeled with their corresponding bootstrap values. The scale bar represents the number of amino acid substitutions per site.

Using the same identification methodology, we conducted analyses in species including *Dendrothrips minowai* [34], *Frankliniella occidentalis* [36], *Frankliniella intonsa* [40], *Frankliniella fusca* [35], *Odontothrips loti* [37], *Thrips palmi* [38], *Stenchaetothrips biformis* [41], *Thrips tabaci* [39], *Megalurothrips usitatus* (Hainan) [32], *Megalurothrips usitatus* (Zhejiang) [33], and *Acyrthosiphon pisum* [49]. The results revealed that the number of OBPs identified ranges from 10 to 17 across these species (Figure 5A). To assess the phylogenetic relevance between *Thrips hawaiiensis* OBPs and other OBPs, all OBPs were aligned to generate unrooted trees. As shown in Figure 5B, the OBPs from the same species (Zhejiang and Hainan populations) were tightly clustered, whereas those from other thrips species were interspersed, a pattern likely attributable to their close phylogenetic relationship within the same family, Thripidae.

### 3.4. Expression Patterns of T. hawaiiensis OBP Genes

**Figure 6:**
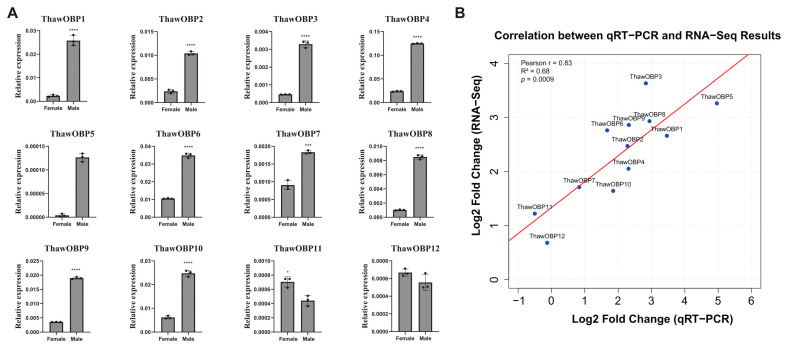
Validation of gene expression profiles. (**A**) Expression profile of OBP genes in female and male adults of *Thrips hawaiiensis* by RT-qPCR. Asterisks denote statistically significant differences between sexes as determined by Student’s *t*-test (* *p* < 0.05, *** *p*< 0.001, **** *p* < 0.0001). (**B**) Correlation analysis of the gene expression ratios obtained from RNA-Seq and RT-qPCR data. The RT-qPCR log2values (expression ratios; y-axis) were plotted against the RNA-Seq log2 values (x-axis). The Pearson correlation coefficient (r) is given in the plot, and the circle indicates the extremely significant difference at *p* < 0.01.

The transcriptomic analysis was conducted using a comprehensive reference-based pipeline. Raw sequencing reads were quality-trimmed and adapter-removed using Trimmomatic (v0.39) [50] to ensure data quality for downstream analyses. Subsequently, the processed reads were aligned to the reference genome using HISAT2 (v2.2.1) [51], a splice-aware aligner optimized for RNA-seq data. Finally, gene-level read counts were quantified from the aligned reads using HTSeq-count (v2.0.2) [52] with default union-counting mode, generating a count matrix for subsequent differential expression analysis. To validate the gene expression profiles obtained from transcriptome sequencing, we selected 12 OBP genes in *Thrips hawaiiensis* for confirmation by RT-qPCR. The results demonstrated that 10 of these genes exhibited male-biased expression (Figure 6A), consistent with transcriptomic predictions. A strong correlation (Pearson correlation coefficient r = 0.83) was observed between the RNA-Seq and RT-qPCR results, validating the reliability of the transcriptomic data (Figure 6B). The male-biased expression patterns were consistent across both methods for most genes, except for ThawOBP11 and ThawOBP12. For these two genes, a discrepancy was noted: they showed minimal expression bias by RT-qPCR (Log2FC ≈ 0 or negative) but were indicated as low-level male-biased by RNA-Seq, a divergence potentially due to their low expression levels or technical limitations.

To investigate whether other olfaction-related gene families follow similar patterns, we also identified genes encoding chemosensory proteins (CSPs) (PF03392.9) using the same pipeline. A total of 11 CSP genes were identified in the *T. hawaiiensis* genome. The expression patterns of the 11 identified CSP genes were analyzed based on transcriptome sequencing and validated by RT-PCR. Transcriptomic data revealed that eight genes were significantly upregulated in males compared to females of *T. hawaiiensis* (Figure S1A). The RT-qPCR validation confirmed the accuracy of the transcriptomic expression profiles (Figure S1B). All primers used in this study are listed in Table S1. The results demonstrate that over half of the genes show significantly higher expression in males, consistent with the expression pattern observed for OBP genes.

## 4. Discussion

The identification of olfactory genes is fundamental to elucidating the molecular mechanisms of olfaction. In many insect species, odorant-binding proteins (OBPs) have been identified through transcriptomic and genomic analyses. Notably, studies have reported 51 OBP genes in *Drosophila melanogaster* [53], 65 in *Anopheles gambiae* [54], 64 in *Aedes aegypti* [54], 53 in *Culex quinquefasciatus* [54], 44 in *Bombyx mori* [55], and 50 in *Tribolium castaneum* [56]. In the present study, by analyzing our previously published genome data, we expanded the repertoire of OBPs in the insect species *Thrips hawaiiensis* to 12. While the 12 OBPs in *T. hawaiiensis* are substantially fewer than those in model insects such as *D. melanogaster*, this number is comparable to other thrips species, such as *M. usitatus* (14,17), *F. occidentalis* (10), *T. palmi* (15), *F. intonsa* (15), and *O. loti* (13) (Figure 5A). The number of OBP genes identified in other insects is significantly larger than that in thrips. This comparative reduction in thrips could be due to a simplified olfactory system or a unique evolutionary trajectory. Furthermore, phylogenetic analysis revealed that ThawOBP11 and ThawOBP12 cluster into a clade and exhibit the highest degree of sequence similarity (Figure 3A), a finding supported by sequence alignment (Figure 2).

For adult thrips, the most critical behaviors are host plant seeking, mating, and reproduction. These behaviors involve the extensive detection of both plant volatiles and thrips pheromones [57,58]. Male adults of both thrips species synthesize an aggregation pheromone detectable by both sexes that mediates attraction [59,60]. Consequently, these genes likely play a role in mediating the detection of both plant volatiles and the male-derived aggregation pheromone in *Thrips hawaiiensis*. Extensive research has demonstrated that odorant exposure can alter the expression levels of OBPs [61,62,63]. Through in vitro and in vivo functional assays in *Bactrocera dorsalis*, OBP83g-2 was identified as a key OBP involved in *γ*-octalactone perception, which was further confirmed to play a significant role in *γ*-octalactone-mediated oviposition behavior [64]. In *Hyphantria cunea*, *OBP2* plays a crucial role in guiding larvae toward food sources that contain adult sex pheromones [65]. While the knockdown of *SfruOBP18* did not impair larval survival or development, our combined RNAi and bioassay approach uncovered its critical function in conferring tolerance to multiple insecticides. This suggests a novel and non-canonical role for *SfruOBP18* in insecticide susceptibility in *Spodoptera frugiperda* [66]. In our study, gene expression pattern analysis shows strong male-specific expression of OBP and CSP genes in *T. hawaiiensis* (Figure 6A and Figure S1). This pattern, also reported for OBPs and CSPs in *M. usitatus*, *Frankliniella occidentalis, and Frankliniella intonsa* [26,30], further supports their functional role in male-driven behaviors such as mate location and foraging. In experiments with *Rhynchophorus ferrugineus*, females injected with OBP-dsRNA showed a significant decrease in the expression of both *RferOBP3* and *RferOBP1768*, which led to impaired perception of the odorants trans-2,4-nonadienal and trans-2-nonenal [67]. Docking results suggested a role for *FoccOBP4*/*FintOBP4*, *FoccOBP6*/*FintOBP6*, and *FoccCSP2*/*FintCSP2* in transporting the major pheromone neryl (*S*)-2-methylbutanoate, with *FoccOBP6*/*FintOBP6*, *FoccCSP2*/*FintCSP2*, and *FoccCSP3*/*FintCSP3* also implicated in binding the minor component (*R*)-lavandulyl acetate [30]. Based on these findings, we hypothesize that the majority of OBP and CSP genes in *T. hawaiiensis* are expressed at higher levels in males than in females, suggesting that males may possess a heightened sensitivity to environmental odors.

## 5. Conclusions

In summary, we report the first genome-wide identification of odorant-binding protein (OBP) genes and chemosensory protein (CSP) genes in *Thrips hawaiiensis* and characterize their expression profiles in both female and male *T. hawaiiensis*. This work provides crucial data for elucidating the functions of olfactory proteins in *T. hawaiiensis*, thereby paving the way for novel, targeted management strategies against thrips pests.

## Figures and Tables

**Table 1:** OBP genes’ primer information used for RT-qPCR.

Primer Name	Primer Sequence (5′–3′)	Length (bp)	Melting Temperature (°C)
qThawOBP1	F:TTCTGTTACACGGCCTGCTT	20	55.40
	R:TTTCACAGGGTTCCTTCCCG	20	57.45
qThawOBP2	F:GAGGTCAACGCCAATACGGA	20	57.45
	R:TGGCCATGCACGACTTTTTG	20	55.40
qThawOBP3	F:GGAGCCCAACCTCAACTTCC	20	59.50
	R:GCACCAGGAATTGTCCGAGT	20	57.45
qThawOBP4	F:TGCTACATGAGCTGCGTGAT	20	55.40
	R:AGGCACTTGTTGTGCTGGTA	20	55.40
qThawOBP5	F:GGTGAAGCTGTACGCCGAG	19	59.48
	R:TCGCTGACACACTCGAACAG	20	57.45
qThawOBP6	F:CCTGAAATGCGTCTACCAGC	20	57.45
	R:GCCTTGTCCTGGATGTCCTC	20	59.50
qThawOBP7	F:AGATGCAGGCCGAGAAGATG	20	57.45
	R:ACGTGAGGTAGTTGAACGCA	20	55.40
qThawOBP8	F:GAAAATGATGGCGTGCGTCT	20	55.40
	R:CGATCTCTTTGGCGCAGTTG	20	57.45
qThawOBP9	F:AGGCCAAAGTCATGACCGAC	20	57.45
	R:AAGAAGTCGTAGCCGAGCAC	20	57.45
qThawOBP10	F:CGTCGAGATCGAGAAGAGCG	20	59.50
	R:TAGGCGGACATCATCTCGGA	20	57.45
qThawOBP11	F:ATGCCCTTCACAATAGAAGTCC	22	55.81
	R:ACGTGCCGCGTTAGGATATT	20	55.40
qThawOBP12	F:CGCTGTGGATGCAAGTGATG	20	57.45
	R:AGGTAGCACTTCATGCCGTC	20	57.45
β-actin	F:TACGAGCTTCCCGACGGTCAGGTT	24	62.98
	R:TGAGGGAGCAAGGGCGGTGATTT	23	61.33

**Table 2:** Identification and characteristics of OBPs in *Thrips hawaiiensis*.

Gene Name	Gene ID	Subfamily	Signal Peptide Location	ORF Len (aa)	Acc. Number	E-Value	Identity (%)
ThawOBP1	g2381.t1	Classic	1–28	155	WBW64293.1	1.85 × 10^−74^	80.159
ThawOBP2	g2658.t1	Classic	1–26	148	WBW64300.1	1.37 × 10^−9^	31.373
ThawOBP3	g3012.t1	Minus-C	1–22	233	WBW64295.1	8.28 × 10^−128^	66.953
ThawOBP4	g4642.t1	Classic	1–19	134	WBW64300.1	3.67 × 10^−8^	32.353
ThawOBP5	g6210.t1	Classic	1–19	189	WBW64305.1	4.55 × 10^−114^	86.842
ThawOBP6	g14828.t1	Classic	1–19	137	WBW64300.1	1.37 × 10^−65^	71.545
ThawOBP7	g15016.t1	Classic	1–27	150	WBW64293.1	1.81 × 10^−28^	39.655
ThawOBP8	g15017.t1	Minus-C	1–17	145	WBW64301.1	1.20 × 10^−75^	76.642
ThawOBP9	g15255.t1	Classic	1–22	156	WBW64298.1	6.04 × 10^−84^	71.613
ThawOBP10	g17204.t1	Classic	1–20	134	WBW64300.1	1.01 × 10^−11^	33.333
ThawOBP11	g97.t1	Classic	1–25	137	WBW64300.1	2.57 × 10^−7^	32.353
ThawOBP12	g153.t1	Classic	1–21	133	WBW64300.1	1.91 × 10^−7^	32.353

## Data Availability

The genomic data for *Thrips hawaiiensis* were retrieved from the figshare database under the accession https://doi.org/10.6084/m9.figshare.26125162.

## Supplementary Materials

The following supporting information can be downloaded at https://www.mdpi.com/article/10.3390/biology15020170/s1: Table S1: CSP genes primer information used for RT-qPCR; Figure S1: Expression profiles of CSP genes. (A) A heatmap illustrates the relative expression levels of each CSP gene in female and male adults. (B) Relative expression levels of CSP genes in female and male *Thrips hawaiiensis* adults were assessed using RT-qPCR. Asterisks indicate statistically significant differences between sexes (Student’s *t*-test: * *p* < 0.05, *** *p* < 0.001, and **** *p* < 0.0001).
