# Supplementary figures and images for: Comprehensive Identification and Male-Biased Expression Analysis of Odorant-Binding Protein Genes in the Hawaiian Flower Thrips, Thrips hawaiiensis (Thysanoptera: Thripidae)
Source: Biology (Basel). 2026 Jan 17;15(2):170. doi: 10.3390/biology15020170 (PMC12837457; doi:10.3390/biology15020170)

A

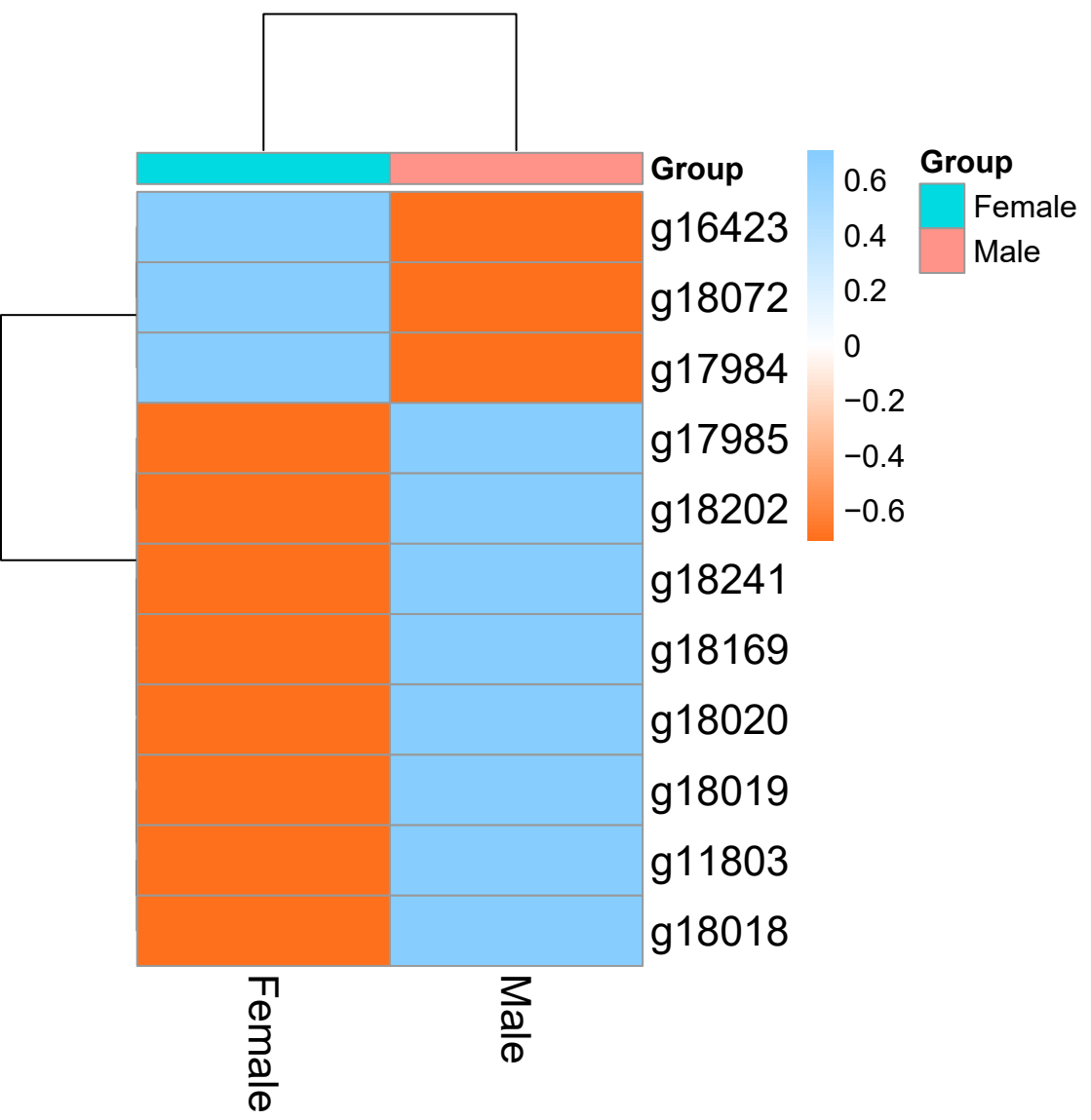

B

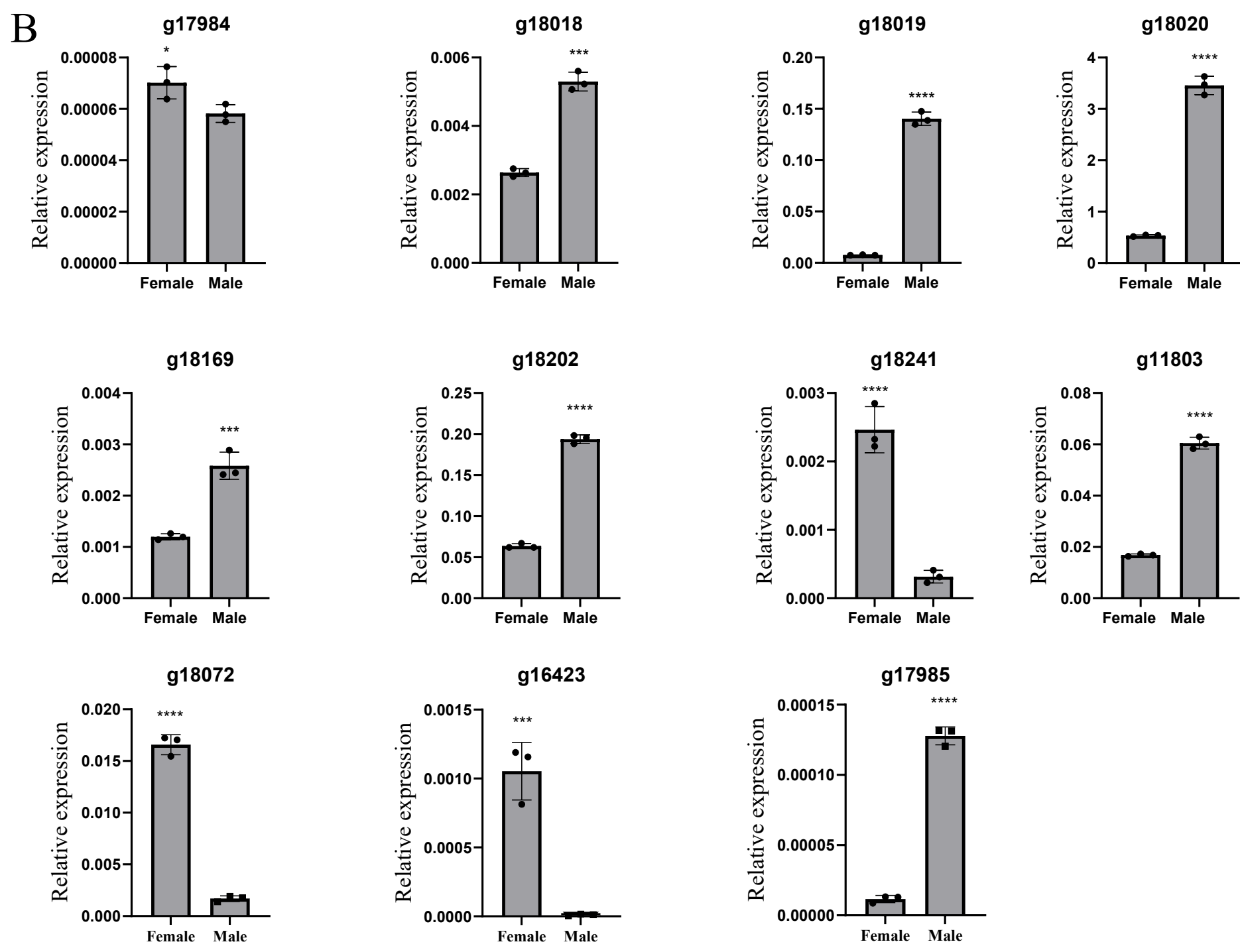

Supplement: Supplementary file 1 [file biology-15-00170-s001.zip › Figure S1.pdf]
